# Hepatic Myelopathy in a Patient with Decompensated Alcoholic Cirrhosis and Portal Colopathy

**DOI:** 10.1155/2012/735906

**Published:** 2012-12-18

**Authors:** Madhumita Premkumar, Avishek Bagchi, Neha Kapoor, Ankit Gupta, Gaurav Maurya, Shubham Vatsya, Siddharth Kapahtia, Premashish Kar

**Affiliations:** Department of Medicine, B. L. Taneja Block, Maulana Azad Medical College and Associated Hospitals, Bahadur Shah Zafar Marg, New Delhi 110002, India

## Abstract

Cirrhotic or hepatic myelopathy is a rare neurological complication of chronic liver disease usually seen in adults and presents as a progressive pure motor spastic paraparesis which is usually associated with overt liver failure and a surgical or spontaneous systemic portocaval shunt. We describe the development of progressive spastic paraparesis, in a patient with alcoholic cirrhosis with portal hypertension and portal colopathy who presented with the first episode of hepatic encephalopathy. The patient had not undergone any shunt procedure.

## 1. Introduction

Hepatic myelopathy (HM) is an insidious onset pure motor spastic paraparesis without sensory or bladder or bowel involvement in patients with liver disease in which the neurological dysfunction cannot be attributed to another disorder. A progressive spastic paraparesis in patients with hepatic failure was first described by Leigh and Card [1], followed by a detailed description of HM  by other authors who observed this rare neurological complication of cirrhosis, especially in patients with portosystemic shunts [2–4]. In India, HM was reported for the first time by Pant et al. who described two cases of spastic paraparesis in patients with liver cirrhosis, one with a spontaneous portocaval shunt and the other with a surgical portocaval anastamosis [5]. The typical clinical presentation of this disorder is of a patient with underlying chronic liver disease, developing progressive pure motor spastic paraparesis with minimal or no sensory deficit and without bowel and bladder involvement. Most patients report prior episodes of hepatic encephalopathy, and in many cases, the development of myelopathy follows the creation of surgical shunts [6–8]. Early and accurate diagnosis of  HM  is important because patients with early stages of the disease can recover following liver transplantation [9]. Neuropathological studies show demyelination in the lateral corticospinal tracts, with varying degrees of axonal loss [2]. Motor-evoked potential studies may be suitable for the early diagnosis of  hepatic  myelopathy, even in patients with preclinical stages of the disease [10]. 

## 2. Case Presentation

A 45-year-old male farmer, hailing from Uttar Pradesh, north India, presented to us with complaints of difficulty in walking due to stiffness of lower limbs for 2 years, associated with weakness. Initially, the weakness was only present on activities like standing from the squatting position or climbing stairs which over a period of a couple of months progressed to noticing slippage of footwear and dragging of feet while walking. He developed a limping gait, with both legs affected symmetrically. To offset this weakness, he reported the use of a walking stick over the last 6 months. There was no history of fasciculations or wasting. Two weeks ago, he noted fever, which rapidly caused prostration. His family reported altered behavior, forgetfulness, lack of attention with disturbed sleep wake cycle. There was no history of bowel and bladder involvement, ocular or vision abnormalities, seizures, diabetes mellitus, hypertension, tuberculosis, trauma, exposure to industrial toxins or radiation, blood or blood component therapy, bleeding disorders, promiscuity, or similar complaints in the family or neighbourhood. His history was significant for one other factor, occasional intake of *Lathyrus sativus* (khesari dal), but not in a quantity or frequency enough to cause lathyrism. He had significant alcohol intake of about 20–30 grams of alcohol at least 3 times a week since the last 15 years. He did not smoke or consume tobacco. General examination revealed normal vitals, average nutrition, pallor, clubbing, and multiple sebaceous cysts involving the back and mild hepatomegaly along with splenomegaly. Other physical markers of liver disease such as icterus, spider angioma, and palmar erythema, were not present. The patient presented with hepatic encephalopathy, with impaired attention and flapping tremors. Spastic paraparesis (Grade III by Medical Research Council scale) was present along with hyperreflexia and bilateral extensor plantar response. He had ankle and patellar clonus. There were no lower motor neuron signs. There were no features of meningeal irritation or cerebellar involvement. Cranial nerve examination and sensory system was normal.

**Figure 1:**
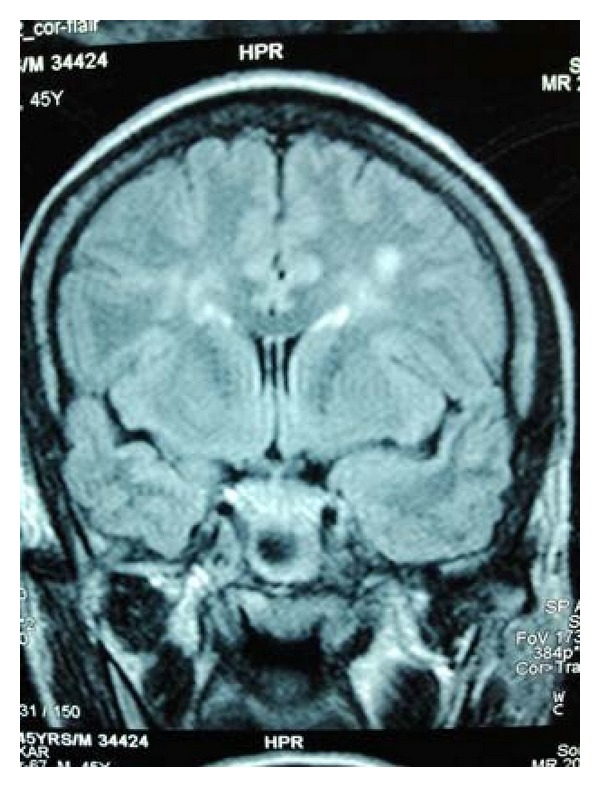
Magnetic resonance image of the brain showing nonspecific white matter changes.

**Figure 2:**
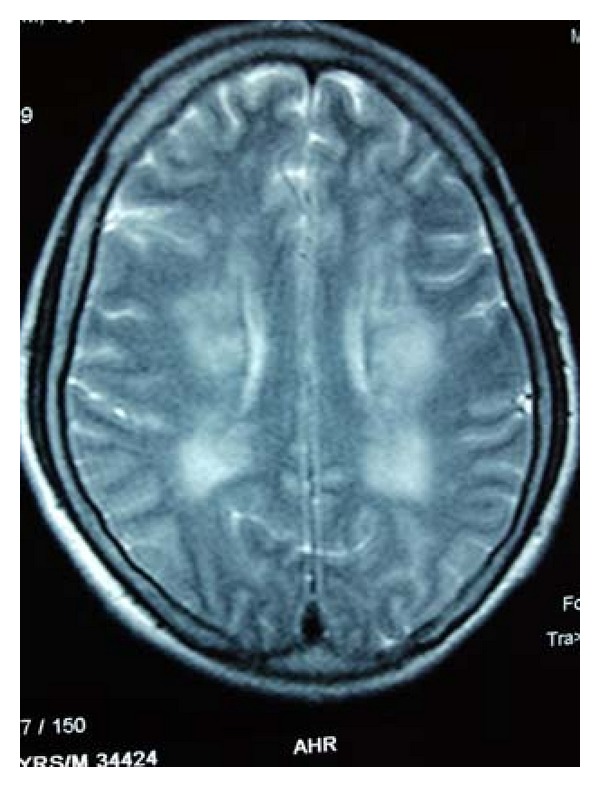
MRI Brain showed nonspecific ischemic changes.

**Figure 3:**
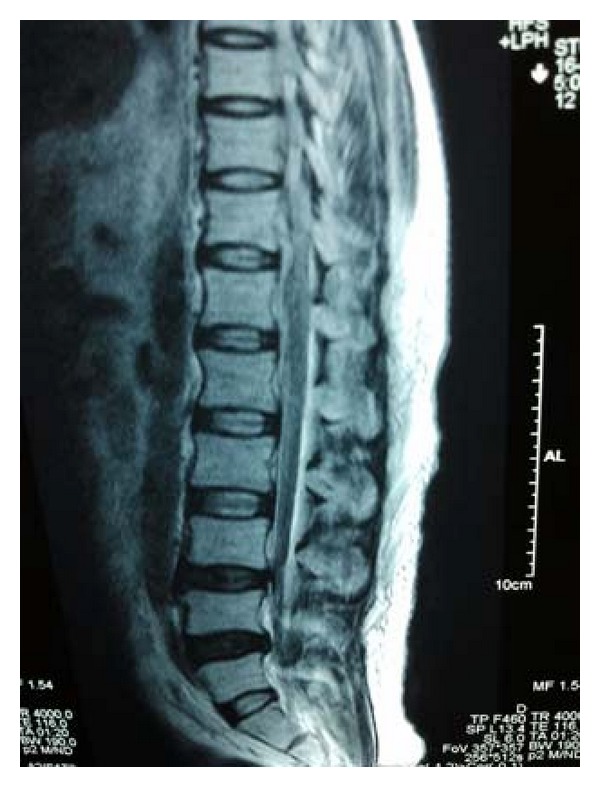
MR imaging of dorsolumbar spine did not reveal any abnormalities, with no evidence of compressive myelopathy.

Investigations revealed mild anemia, mild hyperbilirubinemia, and raised liver enzymes (see Table 1). Cerebrospinal fluid (CSF) examination showed 3 lymphocytes/dL, protein—24 mg%, sugar—67 mg% and was negative for gram's stain, acid fast bacilli, and India ink staining. Ultrasound of the abdomen showed mildly nodular liver with coarsened echotexture with span 13 cm, splenomegaly, a dilated portal vein, and mild ascites. Upper gastrointestinal endoscopy (UGIE) was normal. Electroencephalography revealed background slowing without any spikes suggestive of metabolic encephalopathy. Magnetic resonance imaging (MRI) of the spine and brain showed non specific ischemic changes (see Figures 1, 2, and 3). Patient's serum was nonreactive to hepatitis A, B, C, and E viral markers as well as to HIV I and II. Hb A1c level was within normal limits, which excluded diabetes mellitus.

He developed massive lower gastrointestinal bleed on day 4 and a colonoscopy revealed diffuse portal colopathy with multiple superficial erosions up to the proximal colon. The patient was transfused with fresh frozen plasma but succumbed to the bleed. Postmortem liver biopsy revealed micronodular cirrhosis without steatosis and normal iron stores. 24-hour urinary copper estimation and serum ceruloplasmin were within normal limits. In view of a normal serology, the patient's diagnosis was alcohol-related cirrhosis with portal hypertension with portal colopathy with massive lower GI bleed.

## 3. Discussion

Hepatic myelopathy or porto-systemic myelopathy is a rare neurological complication of chronic liver disease with portal hypertension, usually associated with porto-systemic shunting, and presents as pure motor spastic paraparesis without sensory or sphincter involvement. This is thus a diagnosis of exclusion. The exact pathogenesis of HM is still unclear. Most reported cases are of patients with decompensated liver disease, postliver transplant, or postshunt surgery including TIPSS (transjugular intrahepatic portal systemic shunting) [11]. Other rare reported associations of HM are cases of congenital hepatic fibrosis [12], childhood portal vein thrombosis [8],  and acute hepatitis E [13]. 

Our case is interesting for several reasons. Firstly, this patient presented with a long history of progressive spastic paraparesis with no prior episode of hepatic decompensation. The myelopathy was already advanced before his first episode of encephalopathy. He did not have any prior episodes of gastrointestinal bleeding and the terminal episode of lower gastrointestinal bleeding due to diffuse portal colopathy did not have any antecedent event. Secondly, he had confounding exposure to grass pea, known colloquially as khesari dal (*Lathyrus sativus*). Neurolathyrism is still reported from several states in India, despite extensive awareness programmes about its debilitating neurological effects [14]. The toxin beta oxalyl amino alanine (BOAA) causes lower limb weakness with gluteal atrophy. However, our patient reported infrequent intake of the legume, the weakness was symmetrical, without bladder involvement, and there was no loss of reflexes. The reported toxic dose is about 300 gm of the legume per day for a period of three months [15]. While we were able to exclude certain diagnoses in this case, in view of the long duration of neurological symptoms, the possibility of concomitant predisposing factors like chronic alcoholism and nutrient deficiency cannot be completely ruled out.

It has been hypothesized that the hepatocerebral dysfunction is due to recurrent episodes of hepatic encephalopathy, and prolonged exposure to bypassed nitrogenous waste products such as ammonia, fatty acids, indoles, and mercaptans. These metabolites cause myelin damage resulting in the pathological white matter demyelination in the brain and the spinal cord. The selective predisposition for the motor system has been demonstrated by involvement of the lateral corticospinal tracts in autopsy studies. Other causative factors include nutrient deficiency and deranged liver metabolism. In our patient, the exact cause of myelopathy cannot be explained by the mechanism of recurrent encephalopathy [16].

Conditions which must be excluded include amyotrophic lateral sclerosis, demyelination syndromes like multiple sclerosis and neuromyelitis optica, toxic myelopathy, paraneoplastic syndromes, radiation  myelopathy, HTLV-I associated  myelopathy, and vascular spinal cord disease. The treatment of HM is difficult and the progression of spastic weakness is relentless. Nonetheless, recent reports have suggested that early detection and liver transplantation may improve prognosis in some cases if not all cases [17–20]. 

## 4. Conclusion

Hepatic myelopathy is a rare and debilitating neurological complication of liver failure. Early identification of this disorder and exclusion of other treatable causes is important. The therapeutic potential of liver transplantation for preventing progression and allowing recovery needs to be evaluated further.

## Figures and Tables

**Table 1:** Hematological and biochemical profile of the patient.

Investigations	Day 1	Day 2	Day 4
Hb (g/dL)	11.0	10.5	11.3
Total leukocyte count (cells/dL)	7560	6700	7200
Differential count	P60/L36/M2/E2	P66/L32/E1/M1	P56/L40/E2/M2
Platelet count (cells/*μ*L)	130,000	133,000	140,000
ESR (mm/1st hour)	54		
Urea (mg/dL)	51	36	55
Creatinine (mg/dL)	0.7	0.7	0.8
Na^+^/K^+^ (meq/L)	133/4.1	134/3.5	144/4.6
Total bilirubin/direct bilirubin (mg/dL)	1.8/0.5		1.9/0.9
ALT/AST/ALP (IU/L)	99/243/137		93/143/140
Total protein/albumin (g/dL)	8.0/2.2		
Prothrombin time (test/control (seconds))	15/14		>60/14
Hb A1c (g/dL)		5.1	
